# Juvenile idiopathic arthritis: a single center Lebanese study

**DOI:** 10.1186/1546-0096-12-S1-P179

**Published:** 2014-09-17

**Authors:** Rawane Dagher, Sami Assi

**Affiliations:** 1 Pediatric department, Notre Dame De Secours University Hospital, Byblos, Lebanon

## Introduction

Juvenile Idiopathic Arthritis (JIA) is the most frequent rheumatic disease in childhood. Many variations of JIA are described in different populations. Lebanon is a small middle-eastern country where Pediatric Rheumatology is still emerging as a subspecialty.

## Objectives

To this day, the Lebanese experience concerning JIA is not published. Therefore, the aim of this study is to describe the disease characteristics through a single center series.

## Methods

A retrospective chart review of children who consulted in Pediatric Rheumatology department of Notre Dame De Secours University Hospital between April 2010 and April 2014 was performed. Only patients who met the 2001 classification criteria of the ILAR (International League of Associations for Rheumatology) were included. Epidemiologic and clinical aspects of different types of JIA were studied.

## Results

A total of 66 children were inrolled. The overall sex ratio is 1.The average age at the onset of the disease is 5.2 years (range: 9 months - 14 years). The average age at diagnosis is 5.9 years. The average age at the first visit to the pediatric rheumatologist is 6.6 years. The distribution by types of JIA is as follows: systemic form 23%, persistent oligoarticular form 27%, extended oligoarticular form 4%, polyarticular form with negative Rheumatoid Factor (RF) 24%, enthesitis-related arthritis (ERA) 17%, undifferentiated arthritis 5%. Two sets of siblings are reported including one pair of monozygotic twins. ANA was positive in 15 patients (23%). Only 4 cases of uveitis were observed: 2 in ERA, 1 in persistent oligoarticular form and 1 in extended oligoarticular form. Osteoporosis with vertebral compaction was found in one patient of systemic JIA who had active disease for 4 years. A major growth delay is noted in another patient of systemic JIA who had active disease for more than 6 years.

In this series, diagnosis delay and late referral to the specialist are outlined. Late referral is linked to poor outcome. In comparison to published western data, we found a higher rate of male gender in JIA (50%). Gender distribution particularities are also found in some subtypes of the disease. Systemic and enthesitis related subtypes appear to be more prevalent than in JIA populations described in Europe and North America. Similarly to neighbouring countries, lower rates of ANA positivity and uveitis are noted.

## Conclusion

To the best of our knowledge, this series is the first report on JIA in Lebanon. It shows a distinctive profile of JIA suggesting genetic and environmental roles in disease expression. A major challenge is encountered with late presentation to the pediatric rheumatologist. Larger prospective studies are needed to further elucidate the characteristics of JIA in Lebanon.

## Disclosure of interest

None declared.

